# The processual construction of emotional experience and cultural identity: a qualitative interview study of intangible cultural heritage education in Chinese universities

**DOI:** 10.3389/fpsyg.2026.1878366

**Published:** 2026-07-22

**Authors:** Zixiang Zhou, Ran Xu

**Affiliations:** 1 School of Music and Education, Chuzhou University, Chuzhou, China; 2 School of Music, Anqing Normal University, Anqing, China

**Keywords:** affective experience, constructivist grounded theory, cultural identity, intangible cultural heritage education, sense of community

## Abstract

Within the educational context of intangible cultural heritage (ICH), cultural identity is commonly regarded as a pivotal educational objective. However, a process-oriented, experiential explanation of how emotional experiences participate in the formation and negotiation of cultural identity in educational practice remains underdeveloped. This study investigates Chinese higher education ICH education as its research context. Employing a constructivist grounded theory methodology, we analyzed data from in-depth interviews with 37 experts, university faculty, ICH inheritors, adjunct inheritor-teachers, and students. The findings suggest that participants' cultural identity is not derived from singular cultural contact but rather emerges as a processual experience gradually formed through embodied participation, emotional engagement, and social interaction. During this process, emotional experiences manifest not only as immediate affective responses but also as sustained connective meaning-making within cultural practices, subsequently influencing participants' understanding of cultural belonging, cultural responsibility, and community relationships. The study further observes differentiated narratives across various stakeholders concerning cultural experience, meaning construction, and the articulation of community consciousness, highlighting the distinct context-dependency and non-linear characteristics of cultural identity development. Conceptualizing cultural identity as a processual construct continually negotiated through emotional experiences and social interactions, rather than a fixed or unidirectional experience, this research offers an interpretative perspective for understanding the interrelationships among culture, emotion, and identity in ICH education. The results also underscore the significant roles of the educational context, embodied practice, and multi-stakeholder interaction in the formation of cultural identity.

## 1 Introduction

In the context of deepening globalization and the pervasive embedding of digital media in everyday life, young people's multicultural experiences are increasingly exhibiting deterritorialized and fragmented features. The role of local culture in their identity construction faces new challenges (Zito and Eckersley, 2026). Intangible Cultural Heritage (ICH), as a form of “living cultural practice” (Wu et al., 2025), serves as a crucial trajectory connecting individuals with cultural traditions by integrating embodied experiences (Jiang et al., 2025), affective memories (Zhang and Liu, 2026), and social interactions (Zhang and Ding, 2025) within specific activities. ICH is not merely an issue of preservation; it fundamentally encompasses intergenerational transmission, cultural identity, and community practices (UNESCO, 2003).

Although ICH education has expanded within curriculum systems and practical pedagogy (Koya and Chowdhury, 2020), existing research predominantly focuses on its pedagogical effectiveness or cultural dissemination functions (Orphanidou et al., 2024). The psychological processes through which individuals gradually construct cultural identities via experience, emotion, and meaning-making in specific contexts remain largely understudied. A critical and as yet insufficiently explicated issue is the simultaneous generation of immediate affective responses (Susino et al., 2025) and emotional resonance (Zhang and Liu, 2026) among students engaged in rich cultural experiences. But these emotions do not consistently translate into sustained cultural identities (Prehn et al., 2025), let alone develop into a sense of community with a consciousness of responsibility. The question of why the emotional connections fostered by university faculty and students in experiencing ICH do not naturally evolve into cultural identity (Guerra-Ayala et al., 2026), and how individual-level identity can ascend to a collective “we” consciousness within educational settings, constitute core issues that ICH education practices urgently need to address.

Existing research indicates that affective experiences may contribute to the formation of cultural identity (Dabamona and Cater, 2019); however, the specific trajectories through which they operate within the context of cultural heritage education remain underspecified. Cultural identity is often treated as a singular experience variable (Prehn et al., 2025), neglecting its internal generative trajectories and developmental stages, and lacking a detailed, in-depth study of the “ICH Experience—Cultural Identity” transformation process. On the one hand, at the group level, individual cultural identification does not necessarily lead to the formation of community consciousness (Meca et al., 2021). The transition from “I identify” to “we collectively identify” involves group interaction and the construction of a sense of responsibility through practical engagement, a process that remains systematically unarticulated in the current literature. On the other hand, ICH education is typically embedded within multiple contexts, including classroom instruction, extracurricular activities, field practices, and digital platforms (K. Zhang et al., 2023). How different contextual structures influence identity construction, and how multiple stakeholders, such as university faculty, ICH inheritors, and students, collaborate to jointly transmit ICH culture and education, have also not been adequately explored.

**Figure 1:**
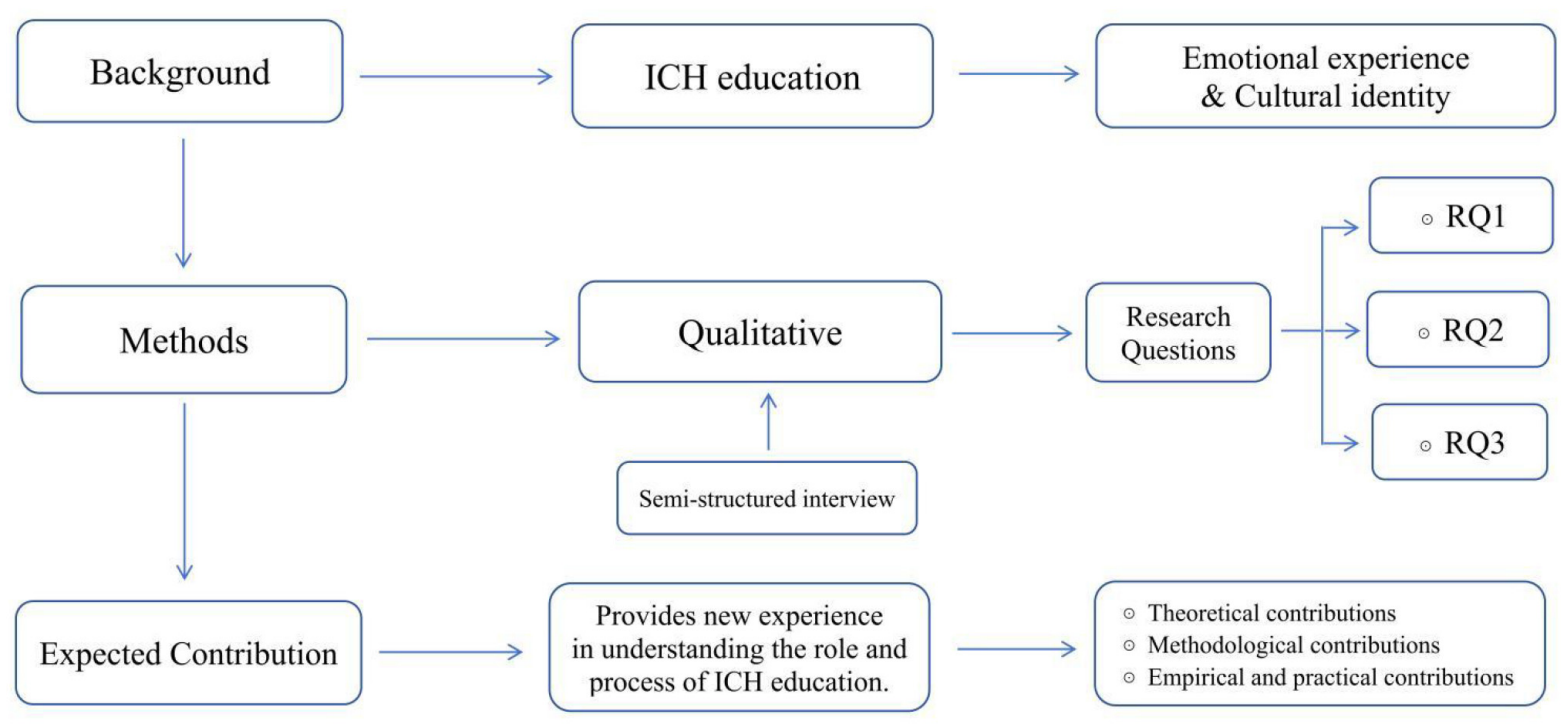
Initial analytical perspectives derived from the literature. This figure illustrates the initial analytical perspectives derived from relevant research within the context of ICH education. It is not intended as a pre-established interpretive framework but rather serves as a source of sensitizing concepts for guiding data collection, interview design, and preliminary analysis.

This study therefore treats affect not as a secondary experience but as a core experiential dimension in the formation of cultural identity, directly responding to the problems outlined above. Based on semi-structured interviews with different groups, and from a process perspective, a multi-level analysis-oriented approach was constructed (Figure 1). Cultural identity is understood as a dynamic process of gradual generation through embodied experience (Ogle et al., 2023), affective resonance (Zhang and Liu, 2026), and meaning construction. This process unfolds through multi-agent interaction and specific contextual conditions, with a particular focus on critical rupture points in identity formation: situations where cultural experience fails to translate into identity, and where identity does not ascend to a sense of collective belonging. We employ a constructivist grounded theory methodology (Charmaz, 2017) to analyze interviews with multiple stakeholders, including experts, university faculty, intangible cultural heritage inheritors, and university students. Our analysis centers on three key research questions:

RQ1: *Within the context of ICH education practices, what is the relationship and transformation process between individual affective experiences and cultural identity?*

RQ2: *In multi-agent interactions, how are affective experiences unfolded between individuals and groups, and how do they form connections?*

RQ3: *Within intangible cultural heritage education practices, what are the differential characteristics of various contexts and affective experiences?*

Through exploring these questions, this research constructs a framework for cultural identity construction (Ward et al., 2025) integrates psychological processes and social contexts. By elevating affect from a dependent variable to an important analytical dimension, we provide a novel empirical foundation and theoretical perspective for understanding the processual role of ICH education. This study makes three primary contributions. First, theoretically, it moves beyond the prevailing paradigm that treats cultural identity as a mere experiential variable, proposing instead a processual framework—anchored in the triad of “embodied experience—affective resonance—meaning construction”—and extending toward collective consciousness—that defines cultural identity as a dynamically generated psycho-social process. Second, analytically, it introduces the perspectives of “contextual embedding” and “multi-agent synergy,” demonstrating how classroom, fieldwork, and practical spaces, together with interactions among teachers, inheritors, and peers, shape identity trajectories—thereby addressing the current neglect of contextual and interactive dynamics. Third, empirically, drawing on multi-agent qualitative data, it identifies two critical junctures of rupture within ICH education, shedding light on the contextual conditions under which identity fails to emerge or deepen. Together, these contributions offer not only a processual framework for understanding cultural identity construction in ICH education but also an actionable analytical toolkit for related research.

## 2 Literature review and theoretical framework

### 2.1 Emotion and cultural identity in ICH education

ICH is a system of cultural expression formed through long-term social practice (Giglitto et al., 2022), carrying the historical memory and values of specific groups (Heredia-Carroza et al., 2021), and serving as a cultural resource for individuals to understand themselves and others. In higher education, ICH is incorporated into curricula and practical training, representing a crucial trajectory for cultural transmission and the cultivation of cultural identity (Koya and Chowdhury, 2020). Research findings suggest that students engaged in ICH courses, craft practices, and fieldwork can enhance their cultural cognition (Xiao et al., 2025) and foster a greater sense of cultural pride and belonging (Hunt et al., 2024). However, existing literature predominantly examines the role of ICH education through the lens of curriculum design (Cheng and Guo, 2024; Liang et al., 2023) and teaching effectiveness, overlooking the emotional experiences of individual participants and their contribution to identity formation (Núñez García et al., 2025; Xue et al., 2019). This results-oriented analytical approach leads to a lack of process-based explanations for “how identity emerges.”

From an affective perspective, emotion is not an individual's private internal state but a relational force that circulates within social interactions and attaches to objects and symbols (Ahmed, 2013). Drawing on Ahmed's work on affect, this study conceptualizes cultural identity as a process shaped by affective investment in practice. Emotions acquire social meaning through sustained connections with objects, symbols, and social relationships. Adopting this perspective, this research understands affect as a crucial experiential dimension that forms relational connections between participants and cultural practices. Consequently, the formation of cultural identity does not stem solely from the acquisition of knowledge or the alignment of values (Balakrishnan et al., 2020), but rather emerges through a gradual process of affective engagement within concrete practices.

In contexts of ICH education, students engage with traditional crafts, performance styles, and associated cultural narratives (Fabry, 2018), potentially eliciting emotional responses and sensory experiences rather than systematic cultural comprehension. Without the activation, transformation, and stabilization of initial affective states during subsequent learning, the development of a sustained cultural identity becomes challenging. Prior research suggests a possible correlation between emotional investment and identity formation experiences, although the evidence remains contingent on specific contextual circumstances. However, from a Community of Practice perspective, cultural learning typically transpires through sustained engagement in shared practices (Wenger, 1998). Existing scholarship predominantly emphasizes the significance of participation and practice in identity construction; yet, the precise processes through which emotional experiences contribute to this process remain inadequately explained. Comprehending ICH education necessitates a shift from a “content transmission” paradigm to a process-oriented view focusing on “affective-meaning generation” to elucidate the developmental trajectories of cultural identity formation.

### 2.2 Embodied experiential cognition and psychological generation processes in cultural identity

Within the field of psychology, cultural identity is typically conceptualized as an individual's sense of belonging and identification with their cultural group (Peterson and Stewart, 2020), encompassing cognitive, emotional, and behavioral dimensions. Social identity theory posits that individuals develop a sense of identification with specific social groups through processes of group categorization and belonging (Hogg, 2016; Tajfel, 1981). This perspective is valuable for understanding the dimension of group affiliation within cultural identity. However, the theory focuses primarily on the structural processes of identity formation, while paying less attention to how identity gradually emerges through concrete cultural practices and emotional experiences.

In recent years, advancements in embodied cognition and emotion research have prompted a reconsideration of identity from experiential and affective perspectives (Deng and Gao, 2025). Embodied cognition theory posits that cognition is not divorced from bodily experience; an individual's comprehension of the world often stems from physical engagement in concrete practices (Hommel, 2015). Within cultural learning practices, culture is not apprehended through abstract symbols but is transformed into perceptible experience through a “learning by doing” process. Furthermore, emotion research highlights that affect is not merely an internal state but circulates within social interactions, shaping identity boundaries (Ahmed, 2013). Cultural learning devoid of emotional involvement is unlikely to solidify into a stable identity.

From a constructivist perspective, individuals do not passively receive pre-existing meanings but actively construct their understanding of the world through the process of interpreting experience (Charmaz, 2006, 2017). Cultural identity can be viewed as a dynamic process propelled by “embodied experience—emotional resonance—meaning construction.” Each stage plays a pivotal role in identity formation. Although this process-oriented perspective offers significant insights into identity, the precise processes through which this unfolds in the context of ICH education within higher education institutions necessitate systematic empirical analysis.

### 2.3 From individual identity to community consciousness

Beyond the individual level, the deepening of cultural identity manifests as the formation of community consciousness (Koya and Chowdhury, 2020). Compared to the sense of belonging and identification emphasized in individual identity, community consciousness prioritizes shared cognition, emotional connection, and responsibility emerging from group interactions (Peterson and Stewart, 2020), centering on the construction of “we.” Social Identity Theory posits that individuals define themselves through group affiliation, but this process does not inherently lead to the generation of community consciousness (Kurakin, 2025). Communities of Practice theory offers a vital supplement for understanding this transition, asserting that identity is forged through sustained engagement in shared practices (Curşeu et al., 2020). Individuals move from peripheral to core participation, ultimately assuming responsibility for and identifying with the group's practices (Koliba and Gajda, 2009). Identity is thus not solely cognitive but also demonstrated through ongoing commitment and emotional investment in collective endeavors (Pan et al., 2019). However, in ICH education research, existing literature often conflates students' interest in or positive attitudes toward ICH with community consciousness (Koya and Chowdhury, 2020), overlooking the complex transformation from “I identify” to “we collectively identify.” This transition inherently involves group interaction, role shifts, and the development of a sense of responsibility; it is a construction process marked by distinct social and procedural characteristics that warrants further investigation.

### 2.4 Situated embedding and practice processes

The generation of cultural identity does not occur in an abstract space but is embedded within concrete social practices (Ozer et al., 2024). Situated learning theory posits (McMains et al., 2023) that learning is an inherently social process embedded in practice, wherein individuals become part of a Community of Practice by engaging in activities within specific contexts and through interactions with others (Lave and Wenger, 1991). In ICH education, various settings, such as classroom instruction, extracurricular clubs, fieldwork, and digital platforms, exhibit significant differences in modes of participation and interaction structures (Xia et al., 2024), diverse impacts exist regarding identification. Practice theory further emphasizes (Raelin, 2026) that culture is not simply transmitted through symbols but is continuously reproduced through daily practices. ICH education is thus not merely the transmission of cultural knowledge but also a process of reproducing and reconstructing cultural practices. Through engagement in skill learning, collective performances, and field investigations, individuals gradually develop an understanding and identification with culture through these practices (Peterson and Stewart, 2020). Moreover, ICH education is inherently a multi-agent collaborative process. Educators are responsible for knowledge organization and pedagogical guidance; ICH inheritors provide authentic experiences and emotional expression, and peer interaction reinforces group belonging and motivational engagement. This multi-agent interactive structure transforms identity construction from an individual internal psychological process (Guerra-Ayala et al., 2026) into a dynamic process of continuous construction and adjustment within social relationships.

### 2.5 Conceptual clarification of key constructs

To avoid conceptual entanglement, this study further defines and differentiates key terms, including “emotion,” “affect,” “affective experience,” “cultural identity,” and “community consciousness.” Initially, “emotion” refers primarily to immediate affective responses within specific contexts, such as brief psychological states like interest, movedness, curiosity, or resonance. In contrast, “affect” emphasizes a more sustained relational emotional intensity, reflecting the affective investment and meaning-making that individuals develop through cultural practice, embodied engagement, and social interaction (Massumi, 2010). Consequently, “affect” exhibits more processual, relational, and generative characteristics than transient emotions. Building upon this distinction, the “affective experience” explored in this research transcends mere emotional reaction; it denotes the sustained experiential affect participants develop within intangible cultural heritage education practices through embodied engagement, cultural interaction, symbolic immersion, and situational attunement. This experience encompasses not only “what is felt” but also “how cultural meaning is progressively understood and the self is re-evaluated through experience.” At the level of identification, “cultural identity” in this study principally signifies an individual's cognitive and affective adherence to specific cultural traditions, their value meanings, and a sense of cultural belonging, manifesting as a subjective awareness of “I understand and identify with this culture.” “Community consciousness,” conversely, extends beyond individual interests or identification to underscore a shared sense of responsibility, collective engagement, and the mission of cultural transmission that participants gradually cultivate through cultural practice. Its core is not simply cultural preference but a collective awareness of “We jointly preserve and perpetuate this culture.” Therefore, this study conceptualizes cultural identity as a processual experience that is continuously generated and negotiated through embodied participation, affective engagement, and social interaction, rather than a fixed, static, or singular identity experience.

### 2.6 Research gaps, theoretical positioning, and research framework

Based on the preceding analysis, current research on ICH education and cultural identity still presents several deficiencies. Three major gaps are evident in current research: (a) systematic analysis of the identity construction process, especially regarding the nexus of experience, emotion, and meaning, is underdeveloped; (b) the pathway from individual identity to collective belonging remains underexplored, along with an integrative perspective on educational settings and multi-actor interactions; (c) insufficient attention has been paid to possible disruptions and failures throughout the identity-building trajectory.

This study treats affective experience as an important analytical dimension that spans different experiential stages, examining its manifestation in the process of identity formation. From a progressive “micro-meso-macro” perspective, it develops an initial process-oriented analytical perspective centered on “embodied experience—emotional resonance—meaning construction—identity recognition—sense of community.” Cultural identity is conceptualized as a dynamic psychological process that gradually emerges within specific contexts and social interactions (Ward et al., 2025). Through systematic analysis of multi-agent interview data, this research aims to explore the possible processes and contextual conditions of identity construction in ICH education, thereby offering new empirical and explanatory insights into understanding the psychological processes of cultural identity.

## 3 Methods

### 3.1 Research design

This study adopts Constructivist Grounded Theory (CGT) as its core methodological framework (Charmaz, 2006, 2017). Given that the research is not focused on linear relationships between variables but rather on the dynamic process by which cultural identity is continuously generated and evolves within intangible cultural heritage education contexts through participants' experiential practices, emotional interactions, and meaning negotiation (Wu et al., 2025), CGT provides an appropriate methodological foundation for understanding this process. In contrast to traditional approaches that view theory as a discovery of objective reality, CGT emphasizes the co-construction of meaning by the researcher and participants, positing that concepts and theories are progressively generated through continuous interaction between the researcher and data within specific socio-cultural contexts. Consequently, this methodology is particularly well-suited for exploring the complex, contextualized, and processual relationships among emotional experiences, embodied engagement, and cultural identity.

Throughout the research and analysis, the analytical logic of Charmaz's CGT was followed. A progressive sequence of “initial coding—focused coding—theoretical coding” was employed to conduct continuous comparative analysis and theoretical abstraction of the interview data. This iterative process facilitated the gradual development of core categories and their relationships, ultimately leading to the formation of an explanatory framework rather than the pre-validation of existing theoretical models.

### 3.2 Participants and sample

This study employed a purposive sampling strategy (Palinkas et al., 2015) to recruit information-rich participants occupying diverse roles within ICH education practices. By including multiple stakeholder groups with relevant experience, the study sought to capture a broad range of perspectives on the processes of cultural identity construction in ICH education. The research subjects were primarily drawn from three universities and five ICH education practice projects located in Anhui Province, China. The research team initially established contact through project leaders, course instructors, and ICH inheritors. Following an explanation of the research objectives and interview requirements, eligible participants were invited to voluntarily join the study.

Inclusion criteria for participants comprised: (1) direct engagement in ICH education-related activities, such as course instruction, project organization, ICH inheritance practice, or curriculum learning; (2) a sustained period of participation enabling reflection and articulation based on personal experience; and (3) voluntary participation and the signing of informed consent forms.

Exclusion criteria included: (1) a lack of direct ICH education engagement experience; (2) the inability to provide complete interview data; and (3) interview content with low relevance to the research topic.

A total of 37 participants were ultimately included in the analysis, consisting of three experts, five university instructors, three part-time inheritor-instructors, five ICH inheritors, and 21 university students. Different stakeholders assume differentiated roles within the ICH education process: experts provide macro-level cognition and theoretical perspectives, university instructors are responsible for curriculum design and teaching implementation, inheritors undertake cultural practice and experience transmission functions, inheritors-instructors bridge the educational and ICH practice domains, and students, as learning and participating subjects, directly experience the formation and development of cultural identity. The multi-stakeholder sample structure facilitates an understanding of the dynamic construction of cultural identity from the perspectives of interactive relationships and contextual embedding.

In the process of data collection and analysis, the study further adhered to the principles of theoretical sampling within constructivist grounded theory (Charmaz, 2006; Conlon et al., 2020). The initial sample primarily served to identify significant experiences, key concepts, and their potential relationships within the context of ICH education. As coding and analysis progressed, the research team selectively recruited additional participants based on emergent conceptual categories and their relationships, aiming to enrich category dimensions, test for category differences, and deepen theoretical explanations. The objective of theoretical sampling was not to enlarge the sample size, but rather to facilitate conceptual development and theoretical construction. For instance, during preliminary analysis, the research team observed an insufficient explanation for the progression from individual identity to collective consciousness. Consequently, ICH inheritors and inheritor-teachers were recruited to gain a more profound understanding of the roles played by different actors in the formation of collective consciousness and their interrelationships. The Supplementary data not only assisted the research team in further elucidating emergent categories but also contributed to the ongoing refinement of category properties and relationships. Throughout the data collection and analysis, theoretical saturation was not assessed as a singular endpoint, but rather continually evaluated through ongoing comparative analysis. As the research advanced, when subsequent interviews ceased to generate substantially new categories, properties, or relationships, the added data predominantly served to supplement and deepen existing categories without yielding new theoretical insights. Under such circumstances, the research team concluded that the relevant categories had been adequately developed, theoretical explanations had stabilized, and thus theoretical saturation had been achieved. This continuous evaluative process ultimately informed the decision to cease further participant recruitment.

### 3.3 Data collection

This study primarily employed semi-structured interviews for data collection (Adeoye-Olatunde and Olenik, 2021), with the interview protocol established through the review of five domain experts. Prior to conducting interviews, participants were thoroughly apprised of the research objectives and their potential implications (Robinson, 2023). The protocol was finalized after three pre-interviews (Appendix 1).

The interview component of this study was collaboratively executed by Zixiang Zhou and Ran Xu. Both researchers possess extensive engagement with the field of ICH education in higher education institutions, coupled with substantial experience in research, learning, and practical application within this domain. Furthermore, they have undergone specialized training in qualitative interviewing techniques. Throughout the data collection process, the researchers maintained an open, respectful, and reflexive research stance. By encouraging participants to fully articulate their experiences and perspectives, and by consistently attending to the researcher-participant interaction dynamics, they endeavored to minimize the influence of pre-existing assumptions on both data generation and interpretive processes. Data collection took place in Anhui Province, China, from March 27 to April 10, 2026, yielding a corpus of 37 interview datasets, with each interview session lasting approximately 30–45 min. Participants were recruited through a combination of purposive and theoretical sampling strategies. During data collection, 24 participants underwent synchronous online or face-to-face semi-structured interviews, while an additional nine participants completed interviews via WeChat voice calls. All 33 oral interview datasets were audio-recorded with participants' informed consent and subsequently transcribed verbatim by the research team. An additional four participants, citing scheduling constraints, were unable to complete real-time interviews. Following the provision of an interview guide consistent with the oral interviews, these participants submitted detailed written responses. These written submissions, which addressed the same core inquiries as the oral interviews, were considered supplementary qualitative data to the interview corpus and were integrated into the subsequent analysis alongside the oral interview data. To ensure comparability across different data sources, The written responses were processed using the same analytical framework and coding procedures applied to the oral interviews. In the data analysis phase, the research team paid close attention to potential disparities in narrative depth, interactional engagement, and modes of expression across the various data sources. Through continuous comparative analysis, they examined the commonalities and divergences between different forms of data to enhance the credibility and robustness of the analytical findings.

The interviews primarily encompassed: (1) participants' specific experiences in engaging with ICH courses and practical activities, with a focus on emotional expression and its developmental trajectory, and the forms and content of embodied experiences; (2) emotional responses and affective shifts during the participation process, and their interplay with cultural experience and identity formation, to explore the development of emotional resonance; (3) participants' understanding and interpretation of the connotations of ICH culture, to analyze the process of meaning construction; (4) participants' recognition and positioning of their own cultural identities, and whether a sense of cultural belonging was formed; (5) experiences during collective activities and interaction processes, to examine the formation of a sense of community; and (6) encountered difficulties, ruptures, or negative experiences during participation, to identify factors hindering identity formation. Interviews were conducted with the informed consent of the participants, audio-recorded throughout, and transcribed verbatim into textual data post-interview, ultimately resulting in approximately 50,000 characters of interview text, providing a robust data foundation for subsequent analysis. During the data collection phase, basic information such as participants' age, gender, academic major, and years of work experience was recorded, adhering to standard experimental data handling protocols, and anonymization was applied during the analysis process (Rodriguez et al., 2022).

### 3.4 Data analysis

During the data analysis phase, the research team concurrently authored analytic memos, systematically documenting observations and reflections on emotional expression, identity shifts, contextual variations, and categorical relationships. These memos served not only to track the evolution of emergent concepts but also facilitated ongoing comparative analysis of participant narratives, thereby progressively deepening the understanding of cultural identity construction. This iterative process led to the formation of explanatory conceptual categories and theoretical linkages. To enhance analytical transparency, Tables 1, 2 delineate the progression from raw quotations to initial coding, focused categories, and overarching themes, illustrating the continuous comparison and theoretical abstraction employed throughout the research. Concurrently, Supplementary material 1 provides representative examples from raw interview data through initial coding to the development of theoretical categories and core themes, showcasing the complete trajectory of theory generation. This study primarily adheres to the analytical logic of constructivist grounded theory as outlined by Charmaz (2017). In its specific implementation, the research adopted an analytical pathway of “initial coding—focused coding—theoretical coding.”

(1) Initial coding. The research team initially conducted a line-by-line analysis of the interview transcripts, conceptualizing statements and passages with independent meaning. During the coding process, the team adhered closely to the participants' original expressions while concurrently undertaking preliminary abstraction in relation to the research questions to generate a large number of initial concepts. Through iterative comparative analysis, the team continuously examined similarities and differences across participants, contexts, and data sources to deepen their understanding of conceptual nuances and interrelationships. To enhance the analytical credibility and transparency, a postdoctoral researcher with qualitative research experience was invited, in addition to the two authors, to participate in partial transcript cross-coding and category discussions. This researcher, who did not contribute to the manuscript's writing or theoretical framework development and was therefore not listed as an author, primarily assisted in verifying coding consistency, discussing conceptual boundaries, and augmenting analytical transparency. During the cross-coding phase, the research team engaged in repeated discussions regarding potential discrepancies in coding interpretations, conceptual attributions, and category boundaries. These discussions, coupled with continuous comparison and refinement against the raw data, led to the establishment of a shared understanding of category definitions and their boundaries. This rigorous process further improved the consistency of the coding outcomes and the credibility of the analytical interpretations. To enhance analytical credibility, approximately 30% of the interview transcripts were independently reviewed and cross-coded by the additional qualitative researcher. Coding differences were not treated as reliability statistics but as opportunities for analytical discussion. Discrepancies regarding concept assignment, category boundaries, or interpretation were examined through repeated reference to the original data and resolved through discussion until a shared understanding was reached.
(2) Focused coding. Building upon continuous comparative analysis, the initial codes were categorized, filtered, and integrated. By comparing similarities and differences across different participants, educational contexts, and experiential narratives, relationships between concepts were gradually identified, leading to the formation of higher-level analytical categories. Through iterative comparison and refinement, this study progressively developed key categories such as embodied cultural experience, emotional resonance, cultural meaning construction, identity affirmation, sense of community, multi-actor collaboration, contextual conditions, and rupture processes.
(3) Theoretical coding. The study further integrates the relationships between categories, exploring their intrinsic connections and pathways of influence to form a holistic explanatory framework. The analysis reveals that the construction of cultural identity in intangible cultural heritage education within higher education institutions is not an instantaneous experience but a dynamic process initiated by embodied cultural experiences. This process progresses sequentially through emotional resonance and cultural meaning construction, undergoing continuous development, negotiation, and adjustment within specific contextual conditions and through multi-agent interactions. Concurrently, the research identifies several rupture mechanisms that can impede or interrupt identity development, demonstrating the distinct non-linear characteristics of cultural identity construction. Throughout the analytical process, the research team engaged in iterative comparisons among data points, categories, and between empirical data and theoretical explanations, thereby continuously refining conceptual relationships until no new categories, attributes, or relationships of substantive significance emerged from the data, thus achieving theoretical saturation.

**Table 1:** Interview analysis and conceptual theme refinement.

ID	Representative statements from the original interview	Concept encoding
T02	“Touching the cultural temperature through each stitch and thread.”	Embodied cultural experience
P03	“My grandmother used to sing this too.”	Familial cultural connection
T02,S07	“If I don't sing it, no one will.”	Cultural responsibility awareness
T01, FT02	“The 32 of us sing it together.”	Collective practice
T01,T02	“Code brings culture to life.”	Creative transformation
S02,S06,S17	“Perceiving intangible cultural heritage as antiquated.”	Negative cognition and rupture
S04,S12	“Insufficient classroom time.”	Institutional constraints
S11	“My work has been recognized.”	External feedback reinforcement

**Table 2:** Example of coding raw interview statements to core themes.

Raw quotation	Initial code	Focused category	Core theme
“When performing Huangmei Opera, memories of elders singing opera at home in my childhood invariably arise.” (T02)	Family cultural memory is activated.	Affective resonance	Affective experience
“As I slowly embroider each stitch and thread, I feel as though I am truly immersed in traditional culture.” (T02)	The body is immersed in cultural practices	Embodied engagement	Embodied participation
“Previously, I perceived these elements as distant from us; however, upon genuine engagement, I realize they constitute our inherent culture.” (S05, S08)	Sense of cultural belonging is enhanced	Identity negotiation	Cultural identity
“If young people do not learn these skills, there is a risk that no one will possess them in the future.” (FT03)	Perception of responsibility for cultural transmission	Collective sense of responsibility	Community consciousness
“During rehearsals in the classroom, the collective experience fosters a sense of shared endeavor, as if we are not learning in isolation but collectively accomplishing a meaningful undertaking.” (S05, T01, FT02)	Meaningful connections within collective participation	Shared practical experience	Collective participation

### 3.5 Researcher reflexivity

Given the research team's long-standing engagement with ICH education and cultural practices in higher education institutions, the interviewer maintained a neutral and objective stance throughout the interview process. During the analysis, the researchers continuously practiced reflexivity (Gesch-Karamanlidis, 2015), critically examining how their own experiences, value orientations, and prior theoretical understandings might influence data interpretation. In the coding and category formation stages, the research team engaged in reflective and corrective discussions through cross-examination, constant comparison, and memo-writing to mitigate the impact of singular perspectives on the analytical experiences. Furthermore, when addressing sensitive topics, interviewers judiciously considered the appropriateness of their questioning and the potential for eliciting biased responses. Subsequently, they would adapt interview strategies accordingly to ensure the collection of authentic and objective information (Elliott, 2018).

### 3.6 Research reliability, validity, and saturation

To bolster the credibility and rigor of the research findings, this study implemented methodological controls from multiple perspectives (Kallio et al., 2016). Firstly, data triangulation was achieved through multi-source data collection, where the experiences of different groups complemented and corroborated each other, thereby mitigating biases arising from a singular viewpoint. Secondly, during the coding process, the research team conducted cross-analysis of a portion of the texts, reaching consensus through discussion to improve coding consistency and stability. Thirdly, through continuous comparison and iterative revision, the consistency of core categories across different datasets was ensured, and analysis ceased upon reaching theoretical saturation, thereby guaranteeing the adequacy of the conclusions. Furthermore, researchers maintained reflexivity (Gesch-Karamanlidis, 2015) throughout the analysis, critically examining how their own positions and experiences might influence interpretations, thus enhancing research transparency. Throughout the interview and analysis phases, subsequent interviews yielded no novel core categories, offering only marginal elaborations on the relationships among existing concepts. Theoretical saturation was assessed continuously throughout data collection and analysis. As coding progressed, newly collected data repeatedly reinforced existing categories and relationships without generating substantively new concepts, category properties, or theoretical connections. In the later stages of analysis, additional interviews primarily contributed elaboration and refinement rather than conceptual expansion. On this basis, the research team concluded that theoretical saturation had been achieved.

### 3.7 Informed consent and ethical approval

This study was conducted in accordance with the ethical principles of the Declaration of Helsinki (Sheather, 2024). Ethical approval was obtained from the Institutional Review Board of Chuzhou University prior to data collection (Approval No. CZSC2026-013). All participants were informed about the purpose of the study, the voluntary nature of participation, their right to withdraw at any stage, and the intended use of the research data before providing informed consent. For oral interviews, participants also consented to audio recording. To protect confidentiality, all identifying information was removed during transcription, analysis, and reporting. Because the dataset contains qualitative narratives from identifiable stakeholder groups involved in ICH education, the data are not publicly available. Requests for access to de-identified data may be directed to the corresponding author and will be considered in accordance with ethical requirements and participant confidentiality agreements.

## 4 Results

### 4.1 Basic characteristics of interview participants

This study conducted in-depth interviews with three experts, five university teachers, three intangible cultural heritage inheritors/part-time teachers, five intangible cultural heritage inheritors, and 21 university students, yielding substantial interview data. Among the total 37 participants, 21 were male and 16 were female, a gender ratio that to some extent reflects the current demographic structure of individuals involved in the transmission and educational practices within the field of intangible cultural heritage. In terms of age distribution, university students ranged from 19 to 23 years old, while other participants were between 36 and 82 years old, with one senior intangible cultural heritage inheritor being 82 years old (Table 3). Participants were evenly distributed in terms of identity and educational background. The diverse participants, drawing from their own practical experiences, shared genuine feelings regarding embodied experience, cultural perception, and identity construction in the context of learning about intangible cultural heritage. This provided multi-dimensional primary data and ensured sample diversity, thereby enhancing the reliability of the research conclusions.

**Table 3:** Basic information of interview participants.

ID	Identity	Age	Gender	Professional	Years of work	Educational
P01	Expert	58	Male	Professor	25	Master's degree
P02	Expert	42	Male	Professor	15	Master's degree
P03	Expert	52	Female	Professor	23	Master's degree
T01	University teacher	46	Male	Associate professor	17	PhD
T02	University teacher	36	Female	Lecturer	7	PhD
T03	University teacher	48	Male	Associate professor	18	PhD
T04	University teacher	50	Male	Professor	20	PhD
T05	University teacher	38	Male	Lecturer	9	PhD
FT01	ICH inheritor and part-time teacher	45	Male	Level 2	24	Master's degree
FT02	ICH inheritor and part-time teacher	48	Male	Level 2	26	Master's degree
FT03	ICH inheritor and part-time teacher	54	Male	Level 2	30	Bachelor's degree
F01	ICH inheritor	82	Female	Level 1	60	Associate's degree
F02	ICH inheritor	56	Female	Level 1	27	Bachelor's degree
F03	ICH inheritor	60	Male	Level 1	40	Associate's degree
F04	ICH inheritor	66	Male	Level 3	45	Associate's degree
F05	ICH inheritor	60	Female	Level 3	41	Associate's degree
S01	University student	19	Male	–	–	Undergraduate
S02	University student	21	Female	–	–	Undergraduate
S03	University student	21	Female	–	–	Undergraduate
S04	University student	19	Female	–	–	Undergraduate
S05	University student	21	Male	–	–	Undergraduate
S06	University student	20	Female	–	–	Undergraduate
S07	University student	19	Male	–	–	Undergraduate
S08	University student	21	Female	–	–	Undergraduate
S09	University student	21	Male	–	–	Undergraduate
S10	University student	18	Female	–	–	Undergraduate
S11	University student	21	Male	–	–	Undergraduate
S12	University student	21	Male	–	–	Undergraduate
S13	University student	21	Female	–	–	Undergraduate
S14	University student	21	Male	–	–	Undergraduate
S15	University student	21	Female	–	–	Undergraduate
S16	University student	20	Male	–	–	Undergraduate
S17	University student	21	Female	–	–	Undergraduate
S18	University student	19	Female	–	–	Undergraduate
S19	University student	21	Male	–	–	Undergraduate
S20	University student	21	Male	–	–	Undergraduate
S21	University student	20	Female	–	–	Undergraduate

### 4.2 Material analysis and thematic elaboration

#### 4.2.1 Analysis of interview data and thematic refinement

Through the analysis of interview data, this study progressively synthesized the following core themes (Tables 1, 2). Embodied Cultural Experience, corresponding to the original statement “touching the cultural temperature stitch by stitch,” signifies the actor's direct perception of the emotions and values embodied in intangible cultural heritage through bodily practice. Familial Cultural Connection, derived from “My grandmother also knows how to sing,” indicates the emotional ties and cultural lineage formed by the intergenerational transmission of intangible heritage skills within families. Cultural Responsibility Awareness, summarized by the expression “If I don't sing, no one will,” mirrors the inheritor's sense of mission and commitment to cultural survival. Collective Practice, based on “The 32 of us sing together,” highlights the practical form of group collaboration and shared participation in intangible heritage transmission. Creative Transformation, corresponding to “Code makes culture come alive,” refers to the innovative expression and vital dissemination of intangible cultural heritage through modern technological means. Negative cognition, rooted in “thinking intangible heritage is outdated,” reflects the stereotypes and dismissive evaluations held by some groups toward intangible cultural heritage. Institutional Constraints, extracted from “There isn't enough class time,” primarily refers to the restrictions on intangible heritage education posed by institutional aspects such as curriculum scheduling. External Feedback Reinforcement, corresponding to “The work was recognized,” signifies the encouraging role of external positive evaluations on the motivation and drive of intangible heritage learners or inheritors.

#### 4.2.2 Association between interview themes and interviewees

The associations between the coded themes derived from interview materials and the interviewees are as follows:

(1) In the Embodied Experience theme, narratives from university faculty, concurrently appointed inheritors/teachers, and inheritors more frequently involved in physical practice, skill engagement, and on-site immersion. Experts and students, conversely, supplemented these with observations and experiential perceptions. Regarding emotional resonance, expert, concurrently appointed inheritor/teacher, and inheritor interviews more frequently featured discussions of emotional cultural connections, intergenerational memory, and emotional expression. University faculty and students tended to narrate based on classroom experiences and learning processes.
(2) Within the Meaning Construction theme, expert and university faculty narratives focused on the interpretation of cultural values, understanding educational significance, and curriculum translation. Inheritors and concurrently appointed inheritor teachers offered supplementary insights based on practical experience, while students primarily expressed their personal interpretations and learning experiences. In the formation of cultural identity, interviews with experts and inheritors emphasized cultural belonging, cultural responsibility, and identity perpetuation. University faculty, concurrently appointed inheritor teachers, and students, however, more often presented experiences of gradual identity formation and negotiation.
(3) Pertaining to the Community Consciousness theme, narratives from experts, university faculty, and concurrently appointed inheritor teachers primarily addressed cultural collaboration, educational responsibility, and inheritance communities. Inheritors and students, on the other hand, expressed their perspectives from the standpoint of participatory experience and collective interaction. At the behavioral transformation level, interviews with inheritors and students more frequently contained expressions related to sustained participation, active dissemination, and practice continuity. Experts, university faculty, and concurrently appointed inheritor teachers focused more on educational support and cultural guidance functions.
(4) In themes concerning context and multi-agent collaboration, experts and university faculty predominantly discussed institutional, curricular, and educational structural aspects. Concurrently appointed inheritor teachers and inheritors underscored the significance of practical fields and cultural interaction, whereas student narratives centered on participatory perception and learning experiences. Regarding the discussion of cultural discontinuity processes, all groups exhibited considerable attention. However, experts, university faculty, and concurrently appointed inheritor teachers analyzed these from perspectives of educational stratification, weakened dissemination, and difficulties in cultural translation. Students and inheritors, in contrast, articulated their views in relation to generational distance and practical participation challenges.

#### 4.2.3 Core theme extraction

Through continuous comparative analysis and theoretical integration of interview data, this study ultimately identified four interconnected core themes (Table 4). These themes are not independent classifications but collectively illustrate the processual experiences and interpretive logics through which participants construct cultural identity within the context of ICH education.

**Table 4:** Extraction of core themes from tertiary coding.

Number	Core Theme	Sub-theme	Main content
TOPIC 1	Micro-level embodied cultural experience	Embodied experience; behavioral transformation	Section 4.3
TOPIC 2	Meso-level emotional resonance and affective engagement	Emotional resonance; identity formation	Section 4.4
TOPIC 3	Macro-level affective cultural meaning construction	Meaning construction; sense of community; situational features	Section 4.5
TOPIC 4	Emotional disjunction and boundary conditions	Multi-agent collaboration; disruption features	Section 4.6

At the micro-level, embodied cultural experiences serve as a crucial starting point for participants' engagement with and understanding of ICH. Through practical activities such as hands-on operations, on-site observations, artistic performances, and skill acquisition, individuals gain direct sensory and experiential knowledge, fostering a propensity for sustained participation and behavioral transformation. Narratives from university faculty, inheritors, inheritors who also teach, and students alike underscore embodied participation as a significant gateway to cultural learning and identity formation.

At the meso-level, embodied experiences are frequently accompanied by the generation of emotional resonance and affective engagement. Participants commonly reported that factors such as the evocation of cultural memories, the life stories of inheritors, collective participatory activities, and personal achievements elicited strong emotional responses. These emotional experiences not only strengthen the affective connection between individuals and ICH but also further promote the development of understandings related to cultural identity.

At the macro-level, affective engagement gradually evolves into a process of cultural meaning construction. By understanding and interpreting the historical background, cultural value, social function, and contemporary relevance of ICH projects, participants' cultural experiences transcend immediate emotional reactions, leading to a deeper appreciation of cultural belonging, responsibility, and the significance of cultural transmission. Within this process, the formation of a sense of community was repeatedly mentioned, often correlating with the development of collective practices, shared participation, and a sense of cultural responsibility.

The study also revealed that the construction of cultural identity does not always follow a linear progression. Interview data indicated that contextual support, multi-stakeholder collaboration, and sustained practical platforms are crucial conditions for fostering identity development. Conversely, factors such as insufficient emotional investment, a lack of interpretation of cultural meaning, limited participation opportunities, and constraints imposed by the institutional environment can lead to interruptions, weakening, or even ruptures in the identity process. Consequently, affective ruptures and boundary conditions also constitute important dimensions for understanding the construction of cultural identity in ICH education.

**Figure 2:**
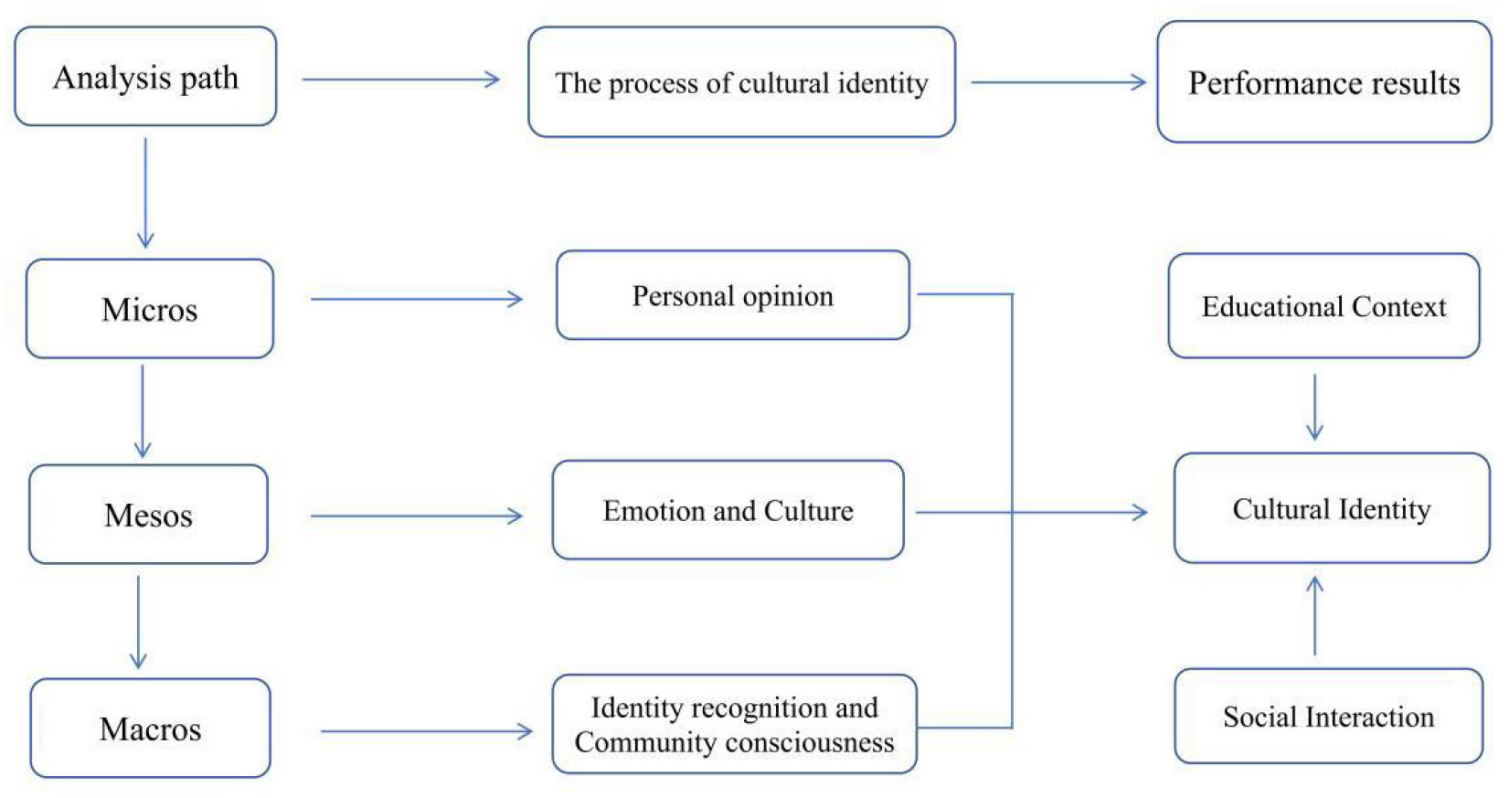
An interpretive framework for cultural identity, formulated through constructivist grounded theory analysis. This figure illustrates the interpretive framework derived from constructivist grounded theory analysis, which was not predetermined but emerged through initial coding, focused coding, theoretical coding, and constant comparative analysis. Research findings indicate that the construction of cultural identity in intangible cultural heritage education is primarily manifested as an interconnected process involving embodied experience at the micro-level, emotion and culture at the meso-level, and identity and community consciousness at the macro-level.

Based on the inherent relationships among the aforementioned core themes, this study further developed a generative explanatory framework (Figure 2) through theoretical coding. This framework is not a pre-established theoretical framework but rather an interpretive framework gradually developed through initial coding, focused coding, theoretical coding, and constant comparative analysis. Within the context of ICH education, the construction of cultural identity is primarily manifested as a dynamic interplay of embodied cultural experience, emotional resonance, cultural meaning construction, identity affirmation, and the development of a sense of community, all of which are continuously influenced by contextual conditions, multi-stakeholder collaboration, and potential rupture processes. Therefore, this framework should be understood as a contextualized explanatory framework derived from participants' experiential narratives, rather than a universally applicable causal framework.

### 4.3 Embodied cultural experience at the micro-level

Embodied cultural experience emerged as an important starting point in participants' accounts of identity construction and was consistently emphasized across different participant groups. This is not a passive “lecture” in a traditional classroom but a process in which learners engage their senses through bodily participation, particularly the hands-on, vocal, ambulatory, and imitative aspects inherent in ICH practices. It is a direct interaction with cultural objects.

From the perspective of experts, embodied experience is posited as an important stage within the entire chain of identification. Experts pointed out that

“*the core of ICH education's influence on youth cultural identity lies in the chain transmission process of ‘immersive cultural experience - affective resonance activation - meaning cognition construction - identity belonging confirmation.'*” (P01)

They emphasized that youth can overcome the “sense of unfamiliarity and distance” from traditional culture only through “direct cultural experience gained via observation, participation, and practice.” Drawing from practical experience in local cultural work, it was explicitly stated that

“*embodied experience is the trigger point; when young students actively engage in hands-on operations, rather than passively listening, bodily memory drives affective involvement*.” (P03)

and that bodily experience itself is considered a necessary prerequisite for activating subsequent affective stages.

Among university faculty, embodied pedagogical design is considered the soul of classroom instruction. Through educational practice, these instructors have distilled a three-stage closed loop: “*field investigation – classroom reconstruction – stage transformation*,” *emphasizing that* “*ICH is not a specimen in a museum, but a living voice with which one can convers*e” (T03). For instance, in digital media courses, “ICH digitization micro-projects” are designed to transform ICH into technical tasks such as “*Anqing ICH H5 interactive page production*” and “*Huangmei Opera vocal visualization*.” This allows students to experience the sense of accomplishment that “*technology brings culture to life*” through coding (T05). Furthermore, emphasis is placed on embedding skill practice and local cultural contexts. The former, in “Music Analysis + Traditional Opera Music Fundamentals + Live Experience,” guides students not only to

“*understand opera music but also to establish intimacy and reverence through singing, listening, analyzing, and performing*.” (T01)

The latter, in the Wangjiang Picking Flowers course, requires students to

“*touch the warmth of culture through every stitch and thread*.” (T02)

The inheritor group directly appeals to the body, employing an experiential form of oral transmission and heart-to-heart instruction. An ICH inheritor who also serves as a part-time teacher summarized,

“*Folk songs cannot only be taught in the piano room*,” (FT02)

advocating for taking students to

“*villages by Chaohu Lake to invite old fishermen to sing authentic sea shanties, letting them hear how the sound of the wind and waves blends with the singing*.” (FT02)

Using Huangmei Opera's body movement instruction as an example, it is stressed to

“*speak no empty words, teach with the body, allowing students to see, hear, and learn*.” (FT03)

This “bodily presence” experiential approach shifts students from abstract symbolic cognition to direct sensory immersion, reserving ample psychological energy for subsequent affective responses.

Student interview narratives further corroborate the important role of embodied experience. During field research participation, one student transformed from

“*merely completing a task to proactively inquiring with artists about wedding customs, labor scenes, and historical stories, becoming completely immersed in the atmosphere of folk culture*,” (S01)

thereby gaining a

“*profound experience of touching the roots of Jianghuai folk culture up close for the first time*.” (S01)

While learning the water sleeves of Huangmei Opera, a university student progressed from “*the sleeve directly winding around their hand*” to experiencing “*releasing the wrist, exerting force at the root of the fingers, and letting the sleeve root drive the sleeve tip, unfolding layer by layer like waves*” (S18), ultimately developing the sensation that “the body speaks for itself” (S15).

In conclusion, the interviews with all groups indicate that embodied cultural experience provides the raw perceptual material and emotional underpinnings for subsequent stages. It constitutes a necessary, though not sufficient, condition for the transformation from “experience to identity.”

### 4.4 Meso-level emotional resonance and affective engagement

Emotional resonance appears to function as an important transitional process subsequent to embodied experience. As noted,

“*Affective engagement may serve as a connective element linking experience and identity-related interpretations; without affective engagement, knowledge remains purely at the informational level*.” (P02)

This study defines emotional resonance as the intermediary nexus through which learners progress from sensory stimulation to internal psychological responses during cultural immersion. Such resonance can manifest as immediate, localized emotional reactions or accumulate and precipitate into a sustained affective disposition toward the cultural object. Through coding analysis, four recurring pathways of emotional resonance were identified: the evocation of local sentiment, the triggering of narratives from inheritors, the experience of collective sonic fields, and the attainment of personal accomplishment (Table 5).

**Table 5:** Four trajectories for the activation of emotional resonance.

Category	Type	Representative interview cases and scenarios
Class I	Evocation of local sentiment (1)	In the Chaohu folk songs, elements such as “silver fish,” “white shrimp,” and “hairy crab” were heard (FT02); “My grandmother used to sing this too” (P03).
Class II	Triggering of inheritor narratives (2)	When performing the ritual song “Cry of the Bride,” the inheritor's voice trembled, her eyes reddened, and “several female attendees present spontaneously wept” (FT02).
Class III	Experience of collective soundscape (3)	During a paired singing exercise in class, one student suddenly became tearful, remarking, “I feel like we are a group standing on a ridge, and it's very moving” (S08/FT02).
Class IV	Attainment of personal accomplishment (4)	University students report experiencing “a profound sense of accomplishment and reverence for traditional culture” upon the completion of their handmade works (S02).

(1) The evocation of local sentiment and familial memories is most prevalent, a finding corroborated by numerous student and inheritor narratives. Upon hearing familiar elements like “Yinyu *(silverfish)*,” “Baixia *(white shrimp)*,” and “Maoxie *(mud crabs)*” in Chaohu folk songs (FT02), students remarked, “*Many said their families eat these, and it immediately felt familiar*” (S03). Some university students, after studying excerpts from “Nv Fuma,” noted,

“*Feng Suzhen's urgency to save her beloved Li is akin to what young people pursue today*.” (S05)

Experts indicate that

“*local knowledge, familial memories, and festive rituals embedded in intangible cultural heritage easily awaken students' affinity for their hometown and traditions, even fostering cultural nostalgia*.” (P02)

The “*discovery of connections to one's own life or memories (e.g.*, ‘*my grandmother used to sing this too*'*)*” is considered a key trigger for emotional resonance (P03).

(2) The authentic stories of inheritors possess an irreplaceable emotional awakening function. When students learned that “Shuband Grinding Songs” were composed by a blind elder artist, “many voices trembled, thinking, ‘*Someone who cannot see sings of the world so beautifully*”' (FT02). Furthermore, during fieldwork on intangible cultural heritage, it was observed that an octogenarian inheritor, while singing “*Cry of the Bride*,” “*had a trembling voice and teary eyes*,” leading “*several girls present to cry on the spot*” (FT02). University instructors also pointed out that

“*inheritors coming into the classroom and recounting experiences passed down through generations makes the skills warm and human*.” (T05)

The emotional transmission carried by authentic life experiences is more direct than abstract cultural advocacy.

(3) The acoustic interplay generated by collective vocalization exerts a unique captivating effect. University instructors recalled,

“*When students were divided into two groups and sang across the classroom, with their voices overlapping, many got goosebumps*.” (T01)

An inheritor who also serves as an adjunct instructor described a similar phenomenon: during a classroom duet,

“*a female student suddenly started crying, saying*, ‘*I feel like we're a group standing on a field ridge, it's so moving*.”' (S08/FT02)

Emotional resonance, induced by collective sound fields, is both physiological and social; the utterance of sound by multiple individuals in the same spatiotemporal context inherently signifies group presence.

(4) The sense of accomplishment and recognition upon completing a work serves as a significant source of emotional resonance. Students expressed “*a sense of accomplishment and reverence for traditional culture*” upon finishing intangible cultural heritage handicrafts (S11). Team members were “*greatly excited*” *when their digital intangible cultural heritage work was* “*seen and liked by staff from the Anqing Municipal Bureau of Culture and Tourism*” (S07). These moments, when actualized and observed, form emotional anchors connecting individuals and culture, and also contribute to understanding the essence of empathetic resonance as an intermediary interpretive process:

“*Students, transitioning from unfamiliarity to recognizing the presence of such elements within their own surroundings… this moment of epiphany often elicits profound emotional fluctuations and subsequent willingness to act*.” (T02)

“*Resonance isn't taught; it happens naturally when sound, stories, and memories converge*.” (FT02)

### 4.5 The construction of affective cultural meaning at the macro-level

The construction of affective cultural meanings emerged as an important interpretive process in participants' narratives, where individuals transform their sensory impressions of culture into rational cognition, signifying a shift from being “affectively touched” to “rationally understanding why they were touched.” Learners are not passive recipients of cultural symbols but actively interpret experiences and establish meaningful connections between cultural objects and their own knowledge structures. Interview and coding analysis suggested four recurring patterns through which affective cultural meanings were constructed.

(1) Deep Understanding of Affective Cultural Connotations. An intangible cultural heritage inheritor serving as a part-time teacher emphasized,

“*Students not only need to know how to sing, but also understand why there are so many* ‘*ya, yo, wei*' *in the lyrics, to grasp that these exclamations are habits of breathing during labor, and to understand that* ‘*dui hua*' *is not just a question-and-answer game, but also a way for young men and women to express affection*.” (FT02)

Through musicological analysis of the melodic ornamentation in Huangmei Opera, students discovered that its “preference for glissando sounds renders it intimate and melodious,” and that its

“*tonal and rhythmic treatment is highly matched with the local dialect, pace of life, and emotional expression styles*.” (S02)

Other students approached it from a literary perspective, comparing the narrative techniques of the lyrics in Nv Fu Ma with the Kong Que Dong Nan Fei ballad from the Han Dynasty Yuefu, recognizing that

“*intangible cultural heritage is not an isolated cultural phenomenon, but a living sample of Chinese folk literature*.” (S17)

These analytical understandings elevate students' cultural experiences from superficial perception to deep cognition.

(2) Affective Meaning Linkages in the Lifeworld. An expert noted in an interview that the key to constructing affective meanings lies in

“*students' ability to establish connections between intangible cultural heritage and their own lifeworlds, such as understanding the ethical values, aesthetic principles, or ecological wisdom behind a certain intangible cultural heritage, thereby forming the cognition that* ‘*this is a part of my cultural DNA*.”' (P01)

In teaching Luocheng folk songs, part-time teachers who are intangible cultural heritage inheritors consciously

“*integrated the classroom with the real rural scenery of Southern Anhui and folk activities, allowing students to sing in authentic environments*,” (FT04)

making the affective connection between folk songs and local life perceptible and knowable.

(3) Decoding Skill Logic and Cultural Codes. Through physical training with water sleeves, students experienced that

“*water sleeves are not mere decoration, but an extension of emotion*” (S08), and “*covering one's face with sleeves when shy, or flinging them away forcefully when angry… each throwing method has a fixed emotional meaning*.” (S11)

Some students derived sociological insights from their investigations into She inkstones, concluding that

“*She inkstones are not just a craft, but a key to understanding the social structure of Huizhou*.” (S09)

Others, through their research on Xuan paper, recognized it as

“*an important carrier of the inheritance of Chinese cultural lineage, a vivid embodiment of the craftsman spirit, and a crystallization of ancient scientific wisdom*.” (S13)

Affective Cultural Translatability and Creative Transformation. In computer-related courses, teachers incorporated a unique “*cultural design statement” segment, “requiring students not only to produce a work, but also to explain the cultural considerations behind each technical choice*” (T04). It was observed that

“*the process of writing this statement compels them to transition from technical implementers to cultural interpreters*.” (T04)

Transformative creation enabled students to internalize meaning through technical practice.

Through interview and coding analysis, we also found that the construction of affective meaning in ICH education can be guided by teachers and inheritors, or generated autonomously by learners through in-depth practice. One expert explicitly pointed out that mere mastery of skills is insufficient for identity formation, stating that

“*guidance is needed to help students understand why this culture is worth protecting together*.” (P03)

Conversely, cases based on student experiences demonstrated that students could independently achieve deep meaning construction through their specialized analytical perspectives. This dual trajectory of guided and autonomous construction emerged repeatedly in participants' narratives during the meaning-construction process. However, meaning construction in ICH education is far from a natural extension of individual identity. Multiple interviewees clearly indicated a risk of rupture between “individual identity” and “community consciousness,” revealing that the critical reason for this interruption often lies in the lack of sustained platforms for collective practice. One inheritor who also serves as a part-time teacher opined, “*The most easily fractured link is from individual identity to community consciousness*” (FT05), attributing this to “*university classrooms primarily focusing on individual learning, lacking continuous collective collaboration scenarios… Post-class, the absence of sustained practical platforms for inheritance makes it difficult to translate classroom collective experiences into stable community consciousness*” (FT05). Experts theoretically posit that individual identity and community consciousness are essentially distinct; the former is characterized by “*I like/recognize this culture*” (P01, P03), while the latter signifies “*I and others together form a group that inherits this culture*” (P01, P03). They emphasized that this transition requires elements such as “group reference, reciprocal commitment, and collective rituals” to be realized.

### 4.6 Emotional ruptures and boundary conditions: cases and contexts of identity failure

Through qualitative analysis of interview data and encoded materials, it is evident that not all ICH experiences successfully translate into affective cultural identity, let alone a sense of community consciousness. A systematic focus on these points of disjuncture constitutes a significant orientation of this research, differentiating it from existing scholarship. Through encoded analysis, the system identified multiple classes of fracturing factors, which aids in understanding the boundary conditions under which the process of identification is interrupted.

(1) Disjunction between Affective Experience and Identity Formation. Specific processes include the superficialization of experience, a lack of guidance in cultural decoding, and the absence of critical triggering events that foster identity. First, concerning the superficialization of experience, an ICH inheritor and adjunct teacher articulated this most profoundly:

“*Many students simply learn two songs in class, watch a video, and find it pleasant and interesting, but this experience is ephemeral… Without repeated listening, singing, and contemplation, the experience quickly dissipates*.” (FT02)

Second, there is a lack of guidance in cultural decoding. The same interviewee pointed out that if instructors do not explain

“*the cultural significance behind the abundant use of interjections like* ‘*ya*,' ‘*yo*,' *and* ‘*wei*' *in the lyrics, it is difficult for students to move from the enjoyment of singing to appreciating the cultural meaning*.” (T04)

Another university instructor attributed the issue to a tendency to “*simplify* ‘*pick-flower*' *embroidery into a craft class, neglecting identity cultivation*” (T03). Furthermore, the evaluative inertia of “prioritizing technique over meaning,” as identified by a university instructor, represents another variant of this disjuncture:

“*When students undertake ICH digitization projects, if a substantial amount of time is spent on cultural research and content design, but the volume of code is insufficient or the interface lacks visual flair, the grades are often unsatisfactory. This leads students to gravitate toward directions with high technical difficulty but shallow cultural substance*.” (T01)

Third, the absence of critical triggering events that foster identity is a significant factor. An ICH inheritor and adjunct teacher emphasized that identity often requires an affective “tipping point,” such as

“*hearing a moving inheritance story, witnessing an elder artist shed tears, or discovering that the folk songs of one's hometown are on the verge of extinction*.” (FT03)

An expert similarly noted that the most susceptible transition is from “affective involvement” to “cognitive connection”;

“*if the experience remains at the level of amusement and fails to guide students in understanding its deeper cultural logic, identity formation is often hindered*.” (P02, P03)

Fourth, negative cognitions and resistant psychological states may also manifest in situations of failed identification. Experts point out that

“*when students perceive ICH education as a form of compulsory cultural indoctrination, it can paradoxically trigger reactance*.” (P02)

“*A considerable number of students do not develop an identity after the experience, or even develop antipathy (considering it ‘uncool' or ‘politicized')*.” (P03)

These cases reveal the boundary conditions of identity formation, yet they are often overlooked. *University students themselves have confessed that prior to engaging with the training of ICH water-sleeve techniques, their perception of traditional arts was limited to a single descriptor:* “*uncool*” (S02, S06, S17).

(2) The affective rupture between individual identity and a sense of collective belonging primarily stems from a deficiency in sustained collective practice scenarios and communal value guidance. Intangible cultural heritage inheritors serving as adjunct instructors have directly observed this phenomenon:

“*Many students can have positive experiences in the classroom and develop personal affection and identification with Luocheng folk songs, but it is difficult for this to naturally transcend to the collective level of shared inheritance*.” (FT04)

Experts have characterized this as a “*structural deficit*” (P02), positing that the leap from individual identification to a collective consciousness requires supporting elements such as group reference, reciprocal commitment, and communal rituals. These elements are precisely and systematically absent within current institutional designs and curriculum frameworks.

1. The affective rupture, attributed to institutional factors, is predominantly manifested in the constraints of class time and a misalignment in evaluation systems. University instructors highlight that

“*intangible cultural heritage education necessitates prolonged immersion, yet university courses often offer only 34 class hours. Just as students begin to develop a connection, the course concludes. The progression from experience to identification and subsequently to a collective consciousness requires repeated engagement and deep participation, which short class hours cannot adequately support*.” (T05)

This instructor also critiqued existing assessment methods that emphasize knowledge recall:

“*If final exams only test the classification of Chaohu folk songs or the vocal characteristics of Huangmei Opera, students will focus their energy on rote memorization rather than on experience and identification*.” (T04)

Furthermore, another university instructor pointed out that “*course evaluations lean toward functional achievement and code quality*” (T02), Students are focusing their efforts on technical virtuosity rather than a deep cultural excavation. This instructor also leveled similar criticisms regarding the institutional environment, where “*evaluation systems prioritize skills over cultural depth*” (T02) and “*emphasize output over cultural literacy*” (T02). These systemic factors fundamentally constrict the temporal, spatial, and psychological investment required for the generation of identification.

## 5 Discussion

This study systematically examines the construction process of cultural identity in higher education ICH education from a processual perspective, based on in-depth interviews with 37 multi-agent participants. In contrast to existing research, which tends to view cultural identity as an educational experience and focuses on evaluating teaching effectiveness, this study finds that cultural identity is not a direct result of a one-off teaching activity (Orphanidou et al., 2024; Prehn et al., 2025). Instead, it is a dynamic experience that is continuously formed, adjusted, and negotiated through embodied participation, emotional involvement, meaning construction, and social interaction. Concurrently, this process is not linear but may be influenced by multiple factors, such as individual experience, educational context, and social support conditions, and can experience interruptions, weakening, or shifts at several critical junctures.

Based on a continuous comparative analysis of respondent narrative data, a process-oriented explanatory framework for the construction of cultural identity in intangible cultural heritage (ICH) education within higher education institutions has been formulated. This framework emphasizes the dynamic interrelationships among embodied cultural experiences, emotional resonance, the construction of cultural meaning, identity affirmation, and a sense of community. It contributes to understanding the significant roles of contextual conditions and disruptive mechanisms in the process of identity development. The research focuses on how cultural identity is experienced, understood, and negotiated within specific educational practices, rather than treating it as a fixed entity or a referent of generalizable value across similar contexts.

### 5.1 The process of emotions transitioning from an ancillary variable to a core generative factor

The findings indicate that participants typically describe the development of cultural identity through affective experiences rather than mere cultural contact. The theoretical contribution of this study lies in elevating affect from a subsidiary variable in cultural identity research to a core driving process throughout its developmental trajectory. This study conceptualizes affect as a form of experience that is both processual and relational (Massumi, 2010). Concurrently, drawing on Ahmed's (2013) discussion of the relationship between affect and social meaning, affective experiences are not isolated but are continually imbued with meaning within cultural practices and social interactions. The empirical data of this study concretize, at an experiential level, the specific operation of this theory within the context of ICH education.

Within the trajectory elucidated by this research, affect does not manifest in a singular form but exhibits differentiated functions across various stages. During the experiential activation phase, affect appears as sensory attraction and contact curiosity at the perceptual level. While university students experience significant difficulty with the dialectal pronunciation when first learning to sing Huangmei Opera, they gradually discover through sentence-by-sentence imitation that they are

“*not just learning a melody, but experiencing someone's emotions*.” (S06)

In the stage of emotional resonance, affect is established through four trajectories: the evocation of local memories, resonance triggered by inheritors' narratives, immersion in collective auditory fields, and the formation of a sense of accomplishment, thereby forging a profound psychological bond between the individual and the culture (Guerra-Ayala et al., 2026). During the identity formation stage, affect progressively crystallizes into more stable cultural pride and a sense of belonging. The feeling of “this is our own culture” emerges from the classroom and is reinforced through social feedback such as performances and recognition. In the phase of community consciousness, affect further expands from emotional responses to cultural objects to an emotional attachment to fellow inheritors and the collective of inheritors itself, signifying a “collective emotion of ‘we inherit together.”' The utterance of a male student described by a university teacher after a group performance,

“*When we sing together, I feel like we are a family*,” (T01, FT02)

exemplifies this budding collective emotion and its expression (Koole et al., 2024).

The findings regarding the multi-layered affective operations resonate with and enrich existing literature on the role of “affective memory” and “collective emotional resonance” in ICH transmission (Susino et al., 2025; Zhang and Liu, 2026), while also providing more nuanced empirical support from a novel perspective. Unlike Zhang and Liu (2026), who primarily discuss the generation of collective emotional resonance within digital exhibition contexts, the findings of this study contribute to understanding the complete conversion processes of emotion from individual sensory experience to collective cohesion in face-to-face, embodied ICH educational settings. This directly addresses the core dilemma posed at the commencement of this research: while immediate emotional responses are frequently observed among students in such educational scenarios, it proves challenging to consistently transform them into sustained cultural identification. The research indicates that the crux of the issue lies not in whether emotions are evoked, but rather in whether they are continually activated and reinforced during subsequent meaning-making and identity-projection phases, and socially anchored through multi-agent interactions.

### 5.2 The transition from experience to identity: meaning construction

This study underscores the independent status of meaning construction as the essential cognitive mediation for the transformation of experience and emotion into identity. In extant literature, the cognitive dimensions of cultural identity have often been broadly subsumed within social identity frameworks of group and self-categorization (Hogg, 2016; Peterson and Stewart, 2020). Through empirical analysis, this research deconstructs the specific components of this cognitive linkage.

Utilizing the four trajectories of meaning construction identified in this study, deep comprehension of cultural connotations, linkage of meaning to lived worlds, cultural decoding of skill logic, and cultural translation and creative transformation, the analysis demonstrated that meaning construction is not a linear transition from reception to identification. Rather, it more closely resembles a practical cognitive endeavor in which learners actively construct interpretive frameworks and enduring personal significance for cultural experiences, leveraging their existing specialized knowledge, life experiences, and analytical capabilities. Meaning construction not only facilitates the establishment of connections between cultural objects and the self but also imbues learners' existing professional competencies with a cultural mission that transcends instrumental rationality.

Furthermore, the co-existence of guided and autonomous construction practices observed in the meaning construction phase enriches theoretical propositions in existing literature advocating for a shift in ICH education from “content delivery” to “affective-meaning generation” (Fabry, 2018; Kurakin, 2025). It indicates practical operational trajectories, highlighting that timely guidance from facilitators and the professional autonomy of learners are mutually indispensable. ICH inheritors serving as part-time educators who employ strategies such as

“*always taking students on fieldwork after their experiences*” (FT02)

effectively integrate experiences into their hearts through real people and events. Essentially, it is within guided construction that appropriate opportunities are provided for students' autonomous meaning-making.

### 5.3 The dual rupture characteristics of intangible cultural heritage education

The systematic identification of “rupture” points in identity formation in this study represents a significant empirical contribution distinct from existing literature. While most research on ICH education focuses on successful identity formation, there is comparatively limited attention paid to contexts where identity fails to materialize or even facilitates negative identifications (Koya and Chowdhury, 2020; Prehn et al., 2025). Our analysis indicates that these ruptures are not random but exhibit structural characteristics at two critical junctures.

The first rupture node lies between experience and identity. Its core reason is the failure of experience to transform into “meaningful cognition.” Factors such as the superficiality of experiences, a lack of cultural decoding, and the absence of trigger events impede the process. This leaves students' cultural engagement at the level of sensory pleasure or skill practice, preventing the construction of deeper meaning. This finding also serves as a theoretical critique of the prevalent “technique-centered” inclination in current ICH education (Shehata et al., 2024), which reduces ICH to mere technical operations. The pedagogical framework pointed out by ICH inheritors,

“*emphasizing technique over emotion, practicing forms without cultural context*,” (F01)

is a significant cause of this rupture.

The second rupture node exists between individual identity and community consciousness, stemming from a lack of structural support. The sustained absence of platforms for collective practice, immersive fields for collective rituals, and continuous organizational forms for cross-curricular student teams within current institutional and curricular frameworks is widespread. As previously discussed, ICH inheritors serving as adjunct instructors characterize this as “*the most easily ruptured link*” (FT01) because “the lack of post-class sustained transmission practice platforms makes it difficult to transform collective classroom experiences into stable community consciousness” (FT03). Experts further posit that

“*the leap from individual identity to collective consciousness requires elements such as group reference, reciprocal commitment, and collective rituals*.” (P03)

However, these elements lack institutionalized cultivation spaces in current ICH education, resulting in a “structural deficit.” The two rupture nodes differ in nature: the former primarily arises from a deficit in the meaning-construction phase, while the latter leans more toward deficiencies at the institutional ecosystem level, thus calling for the introduction of differentiated response strategies.

### 5.4 Contextual embedding and multi-agent collaboration

This research also indicates a profound dependency of identity formation processes on social context and multi-agent interaction. Existing scholarship often situates contextual challenges in ICH education within the frameworks of curriculum design or pedagogical methods (Xia et al., 2024), with insufficient attention paid to the differential regulatory effects of various contextual structures on identity construction. Our findings reveal that diverse contexts, including classrooms, extracurricular clubs, fieldwork, museums, workshops, and digital platforms, not only provide physical spaces for identity formation but also substantially influence the trajectory of identity trajectories through their inherent participatory structures, interactional rules, and power dynamics.

A particularly critical finding focuses on the distinction between “compulsory participation” and “voluntary participation.” During interviews, experts explicitly noted that

“*the identity effects of mandatory curricula and voluntary associations are markedly different*.” (P03)

This also aids in understanding the role distance and emotional distancing students may exhibit in mandatory contexts, contrasting sharply with the active exploration and deep empathy observed in voluntary situations. Museums were characterized as “*safe, open intermediary spaces*” that align with

“*contemporary youth's identity formation logic through autonomous exploration*.” (P03)

This suggests that in ICH educational design, variations in context type should not be overlooked but rather treated as deliberate design variables (Yu and Hu, 2025).

The findings on multi-agent collaboration further reveal that identity construction is not merely an intra-individual psychological process but a social construction process in which multiple actors fulfill distinct roles and engage in reciprocal support across different stages. From the macro-level comprehension offered by expert perspectives, to the knowledge organization and meaning-guidance provided by educators, the affective expression and skill demonstration by inheritors, and finally, the group reference provided by peers, each agent offers irreplaceable structural support at different junctures of the identity formation chain.

### 5.5 Theoretical contributions and practical implications

On a theoretical level, this paper redefines cultural identity as a processual construct unfolding within affective experience. The core contribution of this research lies in the development of a process-oriented analytical framework centered on “embodied experience-affective resonance-meaning construction-identity formation-community consciousness-behavioral transformation.” This framework incorporates situated embedding and multisubject collaboration as modulating dimensions and defines rupture features as boundary conditions of the process. The primary distinctions of this framework from existing literature are threefold. Firstly, it elevates affect from an ancillary variable to a core driving dimension. This offers systematic empirical validation and extension of cultural political affect theory to the level of educational practice (Ahmed, 2013). Secondly, it integrates theoretical resources such as embodied cognition, social identity, and communities of practice from a multidimensional perspective of “process—situation—rupture,” thereby providing a novel integrative analytical framework for research on ICH education. Finally, by identifying rupture features across multiple layers, it furnishes a theoretical toolkit for understanding the experiential boundaries of cultural identity “failure to emerge,” addressing the lacuna in existing research on this blind spot (Guerra-Ayala et al., 2026; Meca et al., 2021).

On a practical level, this study offers several actionable implications for the design and implementation of ICH education. (1) The “meaning decoding” phase, which facilitates the transformation of empathy into identity, should be integrated into curriculum design, providing systematic explanations of cultural context, analysis of craft logic, and reflective discussions guided by instructors. (2) Collective cultural practice platforms, such as group performances, interdisciplinary project collaborations, and long-term fieldwork projects, should be embedded within the curriculum system to serve as institutionalized incubators for the transition from individual identity to community consciousness. (3) By adjusting the curriculum evaluation form to include more process-oriented assessments and affective-identity dimensions (Li and Zhu, 2026), institutional incentives and safeguards for students' deep cultural engagement can be provided. (4) Leveraging the synergistic effects of multiple stakeholders, particularly by systematically integrating the lived experiences and affective expressions of inheritors into pedagogy, is expected to offer more effective triggers for students' affective resonance and meaning construction.

### 5.6 Limitations and future directions

Although this study systematically explored the generation of affective experiences and cultural identity in ICH education contexts from the perspectives of processuality and multi-agent interaction, several limitations warrant further reflection. Firstly, the participants were primarily from Anhui Province, China, with ICH types predominantly focusing on cultural practices such as opera music, traditional crafts, and local folk songs, thus rendering the findings highly context-specific to this region. Differences in ICH forms, educational resources, cultural structures, and intergenerational transmission methods across regions may influence the interaction between affective experiences and cultural identity. Consequently, the explanatory framework proposed in this study requires further enrichment and validation through comparative studies across regions, cultures, and different ICH types. Secondly, this study employed a cross-sectional interview design, meaning the understanding of cultural identity formation processes relied mainly on participants' retrospective narratives. While this approach can reveal participants' meaning-making of their experiences, it may also be influenced by factors such as memory reconstruction, selective recall of contextual elements, and hindsight bias. Therefore, the “processual trajectories” presented in this study should be understood as subjective interpretations of participants' experiences rather than strict temporal causal sequences.

Due to the absence of longitudinal observational data, this study was unable to continuously document the dynamic changes in cultural identity across different stages of learning, practice cycles, and social interactions. Future research incorporating long-term ethnographic observation, periodic interviews, or longitudinal study designs would contribute to a deeper understanding of potential non-linear changes and critical junctures in the identity formation process. Furthermore, the research team's long-standing engagement with ICH education and cultural practices has enhanced their understanding of contextual semantics and cultural nuances, which, however, could also, to some extent, influence data interpretation and theoretical focus. Consequently, throughout the research process, the research team maintained vigilance regarding their own theoretical preconceptions and value stances by continuously employing methods such as analysis memos, cross-discussion, and reflective journaling to minimize the impact of researcher positionality on the analytical experiences.

Although this study encompassed various stakeholders, including experts, university faculty, inheritors, adjunct inheritor-teachers, and students, considerable variations persist within these groups concerning age, gender, artistic discipline, and participation experience. Future research could further enhance the balance and diversity of the sample structure. Concurrently, subsequent studies could build upon this qualitative foundation by operationalizing core categories into measurable dimensions to explore potential structural associations and empirical patterns among different variables.

## 6 Conclusions

This study systematically examines the process of cultural identity formation within ICH education. The findings illuminate the pivotal roles of emotional flow and meaning construction in cultural transmission. Consequently, a processual analytical framework has been formulated, encompassing “embodied experience-emotional resonance-meaning construction-identity formation-community consciousness-behavioral transformation.” Our findings indicate that affect is not monolithic but evolves through four stages: experience activation, affective resonance, identity formation, and a sense of community, progressing from sensory engagement to collective emotional attachment. This dynamic process corroborates and expands theoretical assertions of affect as a relational force. Meaning construction, serving as a critical cognitive mediator, translates fragmented experiences into stable cultural identities via four trajectories: comprehension of cultural connotations, connection to the lifeworld, decoding of artisanal logic, and creative transformation. The synergy between guided and autonomous construction offers actionable avenues for ICH pedagogy.

The research simultaneously identified dual ruptures within intangible cultural heritage education: the former stems from a deficiency in meaning construction, presented through a technocentric approach, while the latter hinders the sustained cultivation of collective emotion due to insufficient institutional support. This observation addresses a gap in existing scholarship concerning the boundary conditions of identity construction. Furthermore, contextual embedding and multi-agent collaboration form the socio-ecological foundation for identity formation, with the differential roles of various participatory contexts and actors offering insights for context-specific pedagogical design.

The theoretical contributions of this study lie in elevating the affective dimension to a core analytical framework, integrating embodied cognition and social identity theories, and constructing an integrated framework that includes disjuncture features and regulatory variables. Practically, it proposes strategies such as enhancing meaning-decoding curriculum design, establishing collective practice platforms, optimizing evaluation modalities, and fostering multi-agent collaboration. Future research could further validate the framework's universality and dynamic characteristics through cross-regional comparisons, longitudinal tracking, and quantitative verification, thereby providing more refined theoretical guidance and practical trajectories for the high-quality development of ICH education.

## Data Availability

The original contributions presented in the study are included in the article/Supplementary material, further inquiries can be directed to the corresponding author.

## Supplementary material

Supplementary material 1 Examples of coding development from raw data to core themes. The interviews selected for this table are representative examples illustrating the process of forming theoretical themes from raw data within a constructivist grounded theory analysis. Initial coding is generated through line-by-line coding, which, on the basis of constant comparative analysis, is further developed into focused coding and theoretical categories, ultimately leading to the distillation of a core theme. This process exemplifies the progressive abstraction from empirical data to theoretical interpretation, thereby enhancing the transparency and traceability of the analytical procedure.

## Appendix 1

Compilation of Interview Groups and Interview Questions

Group 1: University Students

1. What was your most typical, specific experience participating in intangible cultural heritage (ICH) courses or activities? How did you feel at that time?
2. Did this type of experience lead to any affective shifts (e.g., closeness, identification, or alienation) toward the relevant culture? Why?
3. How do you understand the cultural significance behind these experiences? How was this understanding formed?
4. Did these experiences give you a sense of “belonging to this culture”? In what contexts did this feeling emerge?
5. Have you ever experienced an awareness of “we are collectively inheriting a certain culture”? How did this awareness arise?

Group 2: University Teachers

1. In ICH pedagogy, what is your most central instructional design? What type of experience does it aim to stimulate in students?
2. In what contexts do you observe students most readily experiencing affective resonance?
3. What pedagogical approaches do you believe can facilitate students' transition from “understanding culture” to “identifying with culture”?
4. In teaching, where do the critical junctures for students to form a “sense of community” typically occur?
5. What are the most significant obstacles in current pedagogy to this transformative process?

Group 3: Experts

1. Theoretically, what are the core processes by which ICH education influences youth cultural identity?
2. What key psychological processes do students typically undergo when moving from cultural experience to cultural identification?
3. How should “cultural community consciousness” be defined psychologically? What conditions are necessary for its formation?
4. What are the critical missing links in current research concerning the “experience-identification-community” chain?
5. Within the context of globalization, does this process possess cross-cultural explanatory power? In what aspects is this manifested?

Group 4: ICH Inheritors

1. When imparting ICH, what core content do you most hope students will truly experience?
2. During interactions, under what circumstances do students most readily exhibit affective investment or resonance?
3. Have you observed students shifting from “learning skills” to “identifying with culture”? How does this transition occur?
4. In what contexts do students develop the feeling of “this is our culture”?
5. Which teaching or communication methods are least likely to foster identification in students?

Group 5: ICH Inheritors and Part-time Teachers

1. How do you translate ICH experience into pedagogical methods that can evoke genuine student experiences?
2. In your classroom, what factors typically trigger affective resonance in students?
3. Where do the critical shifts from “participation” to “identification” typically occur for students?
4. What do you consider to be the most crucial pedagogical juncture for fostering a “sense of community”?
5. In practice, which link in the chain (experience-identification-community) is most prone to breakage? Why?
