# Supplementary material for: The processual construction of emotional experience and cultural identity: a qualitative interview study of intangible cultural heritage education in Chinese universities
Source: Front Psychol. 2026 Jul 22;17:1878366. doi: 10.3389/fpsyg.2026.1878366 (PMC13437960; doi:10.3389/fpsyg.2026.1878366)
Supplement: Supplementary file 1 [file Data_Sheet_1.docx]

**Supplementary materials 1:**

**Examples of Coding Development from Raw Data to Core Themes**

| **Raw Interview Excerpts (Illustrative Examples)** | **Initial Code** | **Focused Code** | **Theoretical Category** | **Core Theme** | **Participant Group** |
| --- | --- | --- | --- | --- | --- |
| "I encourage students to touch traditional musical instruments and listen directly to performances by heritage inheritors." | Sensory contact | Sensory participation | Embodied engagement | Embodied cultural experience | Expert |
| "Before class, I play recordings of water sounds and traditional work songs collected from the Chaohu lakeside area." | Multisensory stimulation | Context immersion | Embodied engagement | Embodied cultural experience | University Teacher |
| "Students encountered authentic folk songs for the first time during field investigations." | Field exposure | Authentic participation | Embodied engagement | Embodied cultural experience | University Teacher |
| "After putting on traditional opera costumes, students seemed to view themselves differently." | Role immersion | Identity simulation | Bodily practice | Embodied cultural experience | Inheritor Teacher |
| "I personally adjust students’ stage movements and body postures during training." | Demonstration learning | Practice participation | Bodily practice | Embodied cultural experience | Inheritor Teacher |
| "Through repeated rehearsals, the movements gradually became part of their bodily memory." | Repetitive practice | Deep participation | Behavioral transformation | Embodied cultural experience | Student |
| "Students began to search for additional materials and practice singing in local dialects on their own." | Self-directed learning | Behavioral extension | Behavioral transformation | Embodied cultural experience | Student |
| "Many students became deeply moved after listening to the stories shared by heritage inheritors." | Emotional impact | Emotional triggering | Affective engagement | Emotional resonance | Expert |
| "Some students were visibly emotional when they saw elderly performers become tearful." | Emotional contagion | Emotional triggering | Affective engagement | Emotional resonance | University Teacher |
| "Students discovered that similar folk songs also existed in their own hometowns." | Personal connection | Cultural association | Affective engagement | Emotional resonance | Student |
| "Many students found their first experience singing Huangmei Opera in dialect both novel and engaging." | Novel experience | Emotional attraction | Affective engagement | Emotional resonance | Student |
| "Students started asking questions about the historical and cultural background of opera characters." | Curiosity development | Meaning seeking | Affective engagement | Emotional resonance | Inheritor Teacher |
| "Students felt particularly excited and fulfilled when receiving applause after performances." | Achievement experience | Emotional reinforcement | Affective engagement | Emotional resonance | Student |
| "I used to think folk songs were outdated, but now I believe they deserve protection." | Attitude change | Identity transition | Identity recognition | Identity recognition | Student |
| "The transition from learning opera skills to willingly preserving the tradition represented a significant change." | Responsibility awareness | Identity transition | Identity recognition | Identity recognition | Inheritor |
| "Students began actively introducing intangible cultural heritage to their friends and peers." | Cultural expression | Identity presentation | Identity recognition | Identity recognition | University Teacher |
| "I gradually realized that I am also part of the chain of cultural transmission." | Self-positioning | Identity confirmation | Identity recognition | Identity recognition | Student |
| "I feel proud of being able to perform and sing Huangmei Opera." | Cultural pride | Identity affirmation | Identity recognition | Identity recognition | Student |
| "Students began reflecting on why intangible cultural heritage is worth preserving." | Value reflection | Meaning interpretation | Meaning construction | Cultural meaning construction | Expert |
| "They gradually came to understand the historical significance behind traditional opera." | Historical understanding | Meaning interpretation | Meaning construction | Cultural meaning construction | University Teacher |
| "Students realized that intangible cultural heritage carries local cultural memories." | Cultural understanding | Cultural interpretation | Meaning construction | Cultural meaning construction | Expert |
| "Students connected their own personal development with the responsibility of cultural inheritance." | Value internalization | Responsibility construction | Meaning construction | Cultural meaning construction | Student |
| "We composed lyrics together and collaboratively created new folk songs." | Collective creation | Shared participation | Community formation | Community consciousness | University Teacher |
| "Students rehearsed together almost every day in preparation for the performance." | Collective practice | Collaborative engagement | Community formation | Community consciousness | Student |
| "We felt that we belonged to the same group because we were performing the same songs together." | Group belonging | Collective identity | Community formation | Community consciousness | Student |
| "Students came to see themselves as part of a larger chain of cultural inheritance." | Shared responsibility | Collective commitment | Community formation | Community consciousness | Inheritor Teacher |
| "Some students stopped engaging with intangible cultural heritage once the course had ended." | Limited participation | Weak engagement | Rupture process | Emotional rupture and boundary conditions | University Teacher |
| "Although students learned to perform, they often lacked a deeper understanding of the cultural meanings involved." | Meaning deficit | Identity blockage | Rupture process | Emotional rupture and boundary conditions | Inheritor Teacher |
| "Without continuous opportunities for practice, students’ enthusiasm declined rapidly." | Lack of support | Contextual constraints | Boundary condition | Emotional rupture and boundary conditions | Expert |
| "Without a meaningful triggering event, cultural experiences often remain at a superficial level." | Absence of a trigger | Weak emotional activation | Rupture process | Emotional rupture and boundary conditions | University Teacher |

Note: The interviews selected for this table are representative examples illustrating the process of forming theoretical themes from raw data within a constructivist grounded theory analysis. Initial coding is generated through line-by-line coding, which, on the basis of constant comparative analysis, is further developed into focused coding and theoretical categories, ultimately leading to the distillation of a core theme. This process exemplifies the progressive abstraction from empirical data to theoretical interpretation, thereby enhancing the transparency and traceability of the analytical procedure.
